# Post-traumatic stress disorder symptoms among nursing staff who provided direct care to COVID-19 patients: a cross-sectional study

**DOI:** 10.1186/s43045-022-00233-w

**Published:** 2022-09-05

**Authors:** Marina Adel Gabra, Khaled Abd Elmoez Mohammed, Mohammed Nabil Hegazy, Ahmed Elarabi Hendi

**Affiliations:** Department of Psychiatry and Neurology, Faculty of Medicine, Suez Canal University, Ismailia, 41522 Egypt

**Keywords:** COVID-19, Nursing staff, PTSD

## Abstract

**Background:**

The coronavirus disease 2019 (COVID-19) pandemic has led to a major physical and psychological burden on nursing staff who provide patient care in difficult circumstances with persistent exposure to infected patients. This study aimed to assess the prevalence of post-traumatic stress disorder (PTSD) symptoms among nursing staff working during the COVID-19 pandemic and its relationship with different work-related variables. It was designed as a cross-sectional comparative study in which 102 nurses on duty during the past 6 months were enrolled and divided into two groups. The first group included fifty-one COVID-dealing nurses who provided direct patient care to COVID-19 patients (emergency department, isolation zone, and intensive care unit (ICU)), while the second group included fifty-one non-COVID-dealing nurses on duty during the same period but in other hospital units and not providing direct care to COVID-19 patients (inpatient and outpatient wards).

Sociodemographic data, work-related variables, PTSD symptom severity, and diagnosis were all assessed.

**Results:**

The COVID-dealing nurses had significantly less frequent short breaks (*P* = 0.007), inadequate organizational support and compensation (*P* = 0.024), and inadequate time off work (*P* = 0.004) compared to non-COVID-dealing nursing staff. They were also significantly suffering from PTSD compared to second-line staff (*P* = 0.025).

**Conclusions:**

COVID-dealing nurses providing direct care to COVID-19 patients suffered significantly from PTSD with a variety of contributing work-related variables.

## Background

According to the World Health Organization (WHO), continually emerging novel viruses always represent a threat to public health. In 2019, a new coronavirus called severe acute respiratory syndrome coronavirus 2 (SARS-CoV-2) was found in Wuhan, China, which is the largest city in the province of Hubei [1].

Prior to the coronavirus disease 2019 (COVID-19) pandemic, nursing staff always reported high levels of fatigue, little recovery time, and problems with sleep, leading to burnout and decreased psychological well-being [2].

Given the unknown and highly infectious nature of this new virus, the COVID-19 pandemic caused greater physical and psychological difficulties for nursing staff compared to past public health events in the form of increased workload, need for personal protection, and fears of potentially infecting themselves and their families [3].

Seeing patients die, patients’ aggression, dealing with end-of-life care, verbal abuse from family members, feeling overloaded due to inadequate nurse-to-patient ratio, and not being able to save a patient’s life all constitute trauma dimensions that nurses have to continuously face during the pandemic. The symptoms of post-traumatic stress disorder (PTSD) include sleep problems, continuous intrusive thoughts about patients, and irritability. The least common symptoms of secondary traumatic stress are avoidance of people, places, and disturbing dreams about patients [2].

As a result, this study was designed to assess the prevalence of PTSD symptoms among nursing staff working during the COVID-19 pandemic and its relationship with different work-related characteristics to unveil how the nursing population has been affected by the COVID-19 pandemic and formulate efficient evidence-based interventions to support them psychologically.

## Methods

### Participants and procedures

A cross-sectional comparative study which started from April 2021 till October 2021 was done in which 102 nurses on duty during the previous 6 months at Suez Canal University Teaching Hospital were enrolled and divided into two groups:

- Group 1 included 51 COVID-dealing nurses who provided direct patient care to COVID-19 patients; they were working in the following hospital departments:
  1. Emergency department where 60 beds were available for assessing suspected COVID-19 cases on a daily basis
  2. Isolation zone, a building merely assigned to admit confirmed COVID-19 patients with a capacity of 77 beds
  3. Intensive care unit (ICU) where 15 beds were allocated for deteriorating confirmed COVID-19 patients
- Group 2 included 51 non-COVID-dealing nurses on duty during the same period but in other hospital units not directly caring for patients with COVID-19 including 16 inpatient wards and 20 outpatient clinics.

The sample size was calculated according to the proportion of PTSD symptoms among healthcare personnel dealing with COVID-19 patients being 5.7%, absolute error or precision usually equals 10%, and the confidence interval as 1.96. The calculated sample size was 91 participants, but after adding the expected (drop-out) rate (10%), the final sample size was 102 participants [4].

We conducted interviews for all nursing staff who accepted our participation request after excluding those who were on vacation and maternity leaves, nursing administration and leadership, those with a history of pre-existing psychiatric or medical illness, and those with confirmed COVID-19 infection during the past 6 months.

### Tool*s*

The following data were gathered from both study groups:

- Sociodemographic data, including age, sex, marital status, dependent children, and dependent parents
- Work-related data included the unit of practice, years of work experience, employment status, shift length, hours worked per week, frequent short breaks, provision of appropriate work wear, adequate training, organization support and compensation, and time off work
- PTSD symptom prevalence and severity assessments were done using the Short Post-Traumatic Stress Disorder Rating Interview (SPRINT). It consists of eight items that address four core symptoms of PTSD: somatic malaise, stress vulnerability, and impairment in the role and social functioning. A total score of ≥ 14 is considered high on symptom severity, a positive indication for further clinical evaluation for PTSD with Mini-International Neuropsychiatric Interview ( M.I.N.I)

The previous data were collected during the first appointment, and 2 weeks later, the participants who had scores more than 14 on SPRINT were invited to a second appointment for an evaluation with M.I.N.I., a brief structured diagnostic interview for the major psychiatric disorders in DSM-III-R, DSM-IV, and DSM-5, and ICD-10.

### Statistical analysis

The data were coded and imported into the Statistical Package for the Social Sciences (SPSS version 25) software. Data normality of distribution was tested before data analysis. All studied variables were expressed as means, standard deviations, and percentages. The chi-square test, independent samples *t*-test, and logistic regression explained the study results. The results were considered significant if the *P*-value was ≤ 0.05.

## Results

One-third of the COVID-dealing nurses were females; their mean age was 27.00 ± 4.26. Additionally, about one-third of the COVID-dealing nurses had at least one child, and about half of them were dependent parents. There was no statistically significant difference between both study groups regarding sociodemographic data (Table 1).

**Table 1:** Sociodemographic characteristics of the studied population

Variables	Nursing staff		*P*-value
	**COVID-dealing nursing staff (*n* = 51)**	**Non-COVID-dealing nursing staff** **(*n* = 51)**	
**Gender, *n* (%)**			
Male	34 (66.7)	25 (49)	0.071^a^
Female	17 (33.3)	26 (51)	
**Age, mean ± SD**	27.00 ± 4.26	26.69 ± 4.45	0.717^b^
**Social status, *n* (%)**			
Single	29 (56.9)	26 (51)	0.961^a^
Married	23 (43.1)	25 (49)	
**Presence of children, *n* (%)**			
Absent	33 (64.7)	31 (60.8)	0.838^a^
Present	18 (35.3)	20 (39.2)	
One	8 (15.7)	6 (11.8)	
Two	2 (3.9)	5 (9.8)	
Three	1 (2)	0 (0)	
**Dependent parents, *n* (%)**			
No	27 (52.9)	36 (70.6)	0.103^a^
Yes	24 (47.1)	15 (29.4)	

*SD* standard deviation

^a^*P*-values are based on the chi-square test. Statistical significance at *P* ≤ 0.05

^b^*P*-values are based on an independent *t*-test. Statistical significance at *P* ≤ 0.05

One-third of the COVID-dealing nurses was working in the ICU and two-thirds of them were working in the isolation zone. About 60% of the COVID-dealing nurses had working experience of between 3 and 8 years, and 57% had significantly more working hours per week compared to non-COVID-dealing nursing staff (Table 2).

**Table 2:** Work-related characteristics of the studied population

**Work-related characteristics**	**COVID-dealing nursing staff (*n* = 51)**	**Non-COVID-dealing nursing staff (*n* = 51)**	***P*-value**
**Unit of practice, *n* (%)**			
ICU	17 (33.3)	8 (15.7)	< 0.001*
Isolation zone	32 (62.7)	3 (5.9)	
Inpatient non-ICU	0 (0)	28 (54.9)	
ER isolation zone	0 (0)	5 (9.8)	
Intermediate care	0 (0)	5 (9.8)	
Operation room	2 (3.9)	0 (0)	
Clinics	0 (0)	2 (3.9)	
**Years of experience, *n* (%)**			
< 2 years	11 (21.6)	16 (31.4)	0.270
3–8 years	30 (58.8)	20 (39.2)	
9–14 years	4 (7.8)	6 (11.8)	
> 15 years	6 (11.8)	9 (17.6)	
**Employment status, *n* (%)**			
Full time	50 (98)	47 (92.2)	0.169
Part time	1 (2)	4 (7.8)	
**Work hours per week, *n* (%)**			
36 h	3 (6)	11 (21.6)	0.017*
48 h	29 (57)	22 (43.1)	
60 h	13 (25)	8 (15.7)	
72 h	5 (10)	3 (5.9)	
84 h	1 (2)	7 (13.7)	
**Frequent short breaks, *n* (%)**			
No	22 (43.1)	17 (33.3)	0.415
Yes	29 (56.9)	34 (66.7)	
**Providing appropriate work wear, *n* (%)**			
No	16 (31.4)	19 (37.3)	0.677
Yes	35 (68.6)	32 (62.7)	
**Adequate training, *n* (%)**			
Inadequate	12 (23.5)	13 (25.5)	1.00
Adequate	39 (76.5)	38 (74.5)	
**Organization support and compensation, *n* (%)**			
Inadequate	37 (72.5)	37 (72.5)	1.00
Adequate	14 (27.5%)	14 (27.5%)	
**Time off work, *n* (%)**			
Inadequate	29 (56.9)	27 (52.9)	0.842
Adequate	22 (43.1)	24 (47.1)	

*P*-values are based on the chi-square test. Statistical significance at *P* ≤ 0.05

*ICU* intensive care unit, *ER* emergency room

COVID-dealing nurses had significantly higher SPRINT score for the prevalence of PTSD symptoms compared to non-COVID-dealing nurses not providing direct care to COVID-19 patients (18.57 ± 7.69 vs. 14.49 ± 7.01) (*P* = 0.035); 64.7% were having severe symptoms and 51% were diagnosed with PTSD according to M.I.N.I. (Table 3).

**Table 3:** PTSD symptoms, severity, and diagnosis among nursing staff

	**COVID-dealing nursing staff (*n* = 51)**	**Non-COVID-dealing** **nursing staff (*n* = 51)**	***P*-value**
**SPRINT score**			
Mean ± SD	18.57 ± 7.69	14.49 ± 7.01	0.035*^a^
Median (range)	17 (6–36)	15 (0–30)	
**SPRINT severity, *n* (%)**	33 (64.7)	21 (41.2)	0.029*^b^
**PTSD**			
No	25 (49)	37 (72.5)	0.025*^b^
Yes	26 (51)	14 (27.5)	

*SPRINT* Short Post-Traumatic Stress Disorder Rating Interview, *SD* standard deviation, *PTSD* post-traumatic stress disorder

^a^*P*-values are based on the independent *t*-test

^b^*P*-values are based on the chi-square test. Statistical significance at *P* ≤ 0.05

Nursing staff diagnosed with post-traumatic stress disorder had significantly less frequent short breaks (*P* = 0.007), inadequate organizational support and compensation (*P* = 0.024), and inadequate time off work (*P* = 0.004) (Table 4).

**Table 4:** Relationship between work-related characteristics and PTSD among nursing staff

Work-related characteristics	PTSD		*P*-value
	**Absent (*n* = 62)**	**Present (*n* = 40)**	
**Unit of practice, *n* (%)**			
ICU	14 (22.6)	11 (27.5)	0.319
Isolation zone	18 (24.2)	17 (42.5)	
Inpatient non-ICU	20 (32.3)	8 (20)	
ER isolation zone	4 (6.5)	1 (2.5)	
Intermediate care	3 (4.8)	2 (5)	
OR	2 (3.2)	0 (0)	
Clinics	1 (1.6)	1 (2.5)	
**Years of experience, *n* (%)**			
< 2 years	16 (25.8)	11 (27.5)	0.628
3–8 years	28 (45.2)	22 (55)	
9–14 years	7 (11.3)	3 (7.5)	
> 15 years	11 (17.7)	4 (10)	
**Employment status, *n* (%)**			
Full time	59 (95.2)	38 (95)	0.971
Part time	3 (4.8)	2 (5)	
**Hours worked per week**			
36 h	11 (17.7)	3 (7.5)	0.177
48 h	34 (54.8)	17	
60 h	10 (16.1)	4 (10)	
72 h	3 (4.8)	11 (27.5)	
84 h	4 (6.5)	5 (12.5)	
**Frequent short breaks**			
No	17 (27.4)	22 (55)	0.007*
Yes	45 (72)	18 (45)	
**Provision of appropriate work wear**			
No	19 (30.6)	16 (40)	0.394
Yes	43 (69.4)	24 (60)	
**Adequate training**			
Inadequate	13 (21)	12 (30)	0.349
Adequate	49 (79)	28 (70)	
**Organization support and compensation**			
Inadequate	40 (64.5)	34 (85)	0.024*
Adequate	22 (35.5)	6 (15)	
**Time off work**			
Inadequate	27 (43.5)	29 (72.5)	0.004*

*P*-values are based on the chi-square test. Statistical significance at *P* ≤ 0.05

*PTSD* post-traumatic stress disorder

Regression analysis used to assess predictors of PTSD among nursing staff showed a three-time increase in the odds of having PTSD for nursing staff who provide direct patient care to COVID-19 patients compared to non-COVID-dealing nursing staff (*P* = 0.019). Moreover, there was a 2.643-time increase in the odds of having PTSD for nursing staff who did not have frequent short breaks (*P* = 0.036) (Table 5).

**Table 5:** Logistic regression analysis of determinants of PTSD among nursing staff

**Predictors**	**Unstandardized coefficients**		**Odds ratio** **(95% CI)**	***P*-value**
	***B***	**SE**		
**Constant**	− 1.622	1.789		0.365
**Age**	− 0.023	0.057	0.977 (0.874–1.094)	0.690
**Gender**				
Female vs. male (R)	− 0.373	0.503	0.689 (0.257–1.847)	0.459
**Nursing staff**				
Second line vs. first line (R)	1.098	0.468	2.998 (1.196–7.501)	0.019*
**Frequent short breaks**				
Yes vs. no (R)	0.972	0.464	2.643 (1.064–6.563)	0.036*
**Organization support and compensation**				
Adequate vs. inadequate (R)	0.785	0.617	2.192 (0.655–7.338)	0.203
**Time off work**				
Adequate vs. inadequate (R)	0.759	0.519	2.137 (0.773–5.907)	0.143

Statistical significance at *P* ≤ 0.05

## Discussion

This study aimed to assess the PTSD symptoms among nursing staff working during the COVID-19 pandemic and its relationship with different work-related characteristics. Our findings showed that COVID-dealing nurses providing direct care to patients with COVID-19 were three times more likely to have PTSD than non-COVID-dealing nurses not providing direct care to COVID-19 patients.

We found that 64.7% of the COVID-dealing nurses had severe PTSD symptoms, and 51% were diagnosed with PTSD. Less frequent short breaks, inadequate organizational support and compensation, and inadequate time off work were the most work-related characteristics significantly associated with PTSD among COVID-dealing nurses.

Providing care to COVID-19 patients coupled with more time spent in the hospital leads to repeated exposure to trauma, which may have significantly increased the risk of PTSD [5].

Additionally, fear of transmitting the disease to family members, paucity of resources, heavy workloads, caring for potentially rapidly deteriorating patients, seeing colleagues continuously fall sick, and lack of psychological and social support also increase the risk of PTSD [6].

Similarly, a study that assessed the mental health of healthcare workers during the COVID-19 pandemic in Italy revealed that healthcare professionals working in COVID-19 wards reported higher levels of depressive symptoms and post-traumatic stress symptoms compared to those who work in other healthcare units. Also, the authors noted that being an older female is related to higher levels of post-traumatic stress symptoms [7].

Moreover, in China, a mental health survey of medical staff in a tertiary infectious disease hospital for COVID-19 showed that the incidence of anxiety and PTSD is high among medical staff, especially female nurses [8].

Another Chinese online survey carried out on medical health workers during the COVID-19 outbreak reported that medical health workers had a higher prevalence of insomnia, anxiety, depression, somatization, and obsessive–compulsive symptoms compared with non-medical health workers [9].

According to another cross-sectional online survey conducted in Turkey to assess psychological responses of healthcare workers and related factors during the COVID-19 outbreak, being female, being young, being single, having less work experience, and working in frontline jobs were associated with higher scores of stress [10].

Also, several previous studies described acute and post-traumatic stress among healthcare staff working with patients during viral outbreaks, which was related to a variety of sociodemographic and work-related characteristics [11].

Predisposing factors were being women [12, 13], younger [14], parents of dependent children [15], exposed to prolonged quarantine [16], having pre-existing psychological or physical illness [17], and fear of infecting or having an infected family member [18].

Significantly work-related risk factors among nurses were being less experienced, part-time employment, and frustration about the effect of precautionary measures on their ability to do their jobs [19].

Other factors included inadequate staff training, organizational support, compensation, and societal stigma against healthcare workers [20].

Protective factors found by previous studies were being older, having greater clinical experience, frequent short breaks from clinical duties, adequate time off work, family support, adequate training, a supportive work environment, clear communication, and faith in precautionary measures^.^ [21].

Having access to psychologically supportive interventions was also noted to be protective [22]. Although nurses are more vulnerable to psychological distress than other healthcare workers, they are more likely to adhere to infection control procedures [23]. Lastly, seeing infected colleagues getting better, as well as a general drop in disease transmission, improved psychological outcomes [24].

This study highlights the importance of healthcare workers’ mental health caring for patients during a viral outbreak. The COVID-19 pandemic has burdened our nursing population and exacerbated previously existing problems such as post-traumatic stress, so effective interventions including communication, access to adequate personal protective equipment (PPE), more frequent short breaks, adequate rest, and psychological support should be immediately implemented.

## Conclusions

COVID-dealing nurses providing direct care to patients with COVID-19 who are having less frequent short breaks, inadequate organizational support and compensation, and inadequate time off work are more likely to have PTSD compared to non-COVID-dealing nurses not providing direct care to COVID-19 patients.

## Data Availability

All data generated or analyzed during this study are available on request.

## Abbreviations

COVID-19: Coronavirus disease 2019
PTSD: Post-traumatic stress disorder
WHO: World Health Organization
SARS-CoV-2: Severe acute respiratory syndrome coronavirus 2
M.I.N.I: Mini-International Neuropsychiatric Interview
PPE: Personal protective equipment
